# Identification of nine sequence types of the 16S rRNA genes of *Campylobacter jejuni *subsp. *jejuni *isolated from broilers

**DOI:** 10.1186/1751-0147-50-10

**Published:** 2008-05-21

**Authors:** Ingrid Hansson, Marianne Persson, Linda Svensson, Eva Olsson Engvall, Karl-Erik Johansson

**Affiliations:** 1 Department of Bacteriology, National Veterinary Institute, SE-751 89 Uppsala, Sweden; 2 Swedish Zoonosis Center, Department of Disease Control, National Veterinary Institute, SE-751 89 Uppsala, Sweden; 3 Department of Biomedical Sciences and Veterinary Public Health, Swedish University of Agricultural Sciences, SE-750 07 Uppsala, Sweden

## Abstract

**Background:**

Campylobacter is the most commonly reported bacterial cause of enteritis in humans in the EU Member States and other industrialized countries. One significant source of infection is broilers and consumption of undercooked broiler meat. *Campylobacter jejuni *is the *Campylobacter *sp. predominantly found in infected humans and colonized broilers. Sequence analysis of the 16S rRNA gene is very useful for identification of bacteria to genus and species level. The objectives in this study were to determine the degree of intraspecific variation in the 16S rRNA genes of *C. jejuni *and *C. coli *and to determine whether the 16S rRNA sequence types correlated with genotypes generated by PFGE analysis of *Sma*I restricted genomic DNA of the strains.

**Methods:**

The 16S rRNA genes of 45 strains of *C. jejuni *and two *C. coli *strains isolated from broilers were sequenced and compared with 16S rRNA sequences retrieved from the Ribosomal Database Project or GenBank. The strains were also genotyped by PFGE after digestion with *Sma*I.

**Results:**

Sequence analyses of the 16S rRNA genes revealed nine sequence types of the *Campylobacter *strains and the similarities between the different sequence types were in the range 99.6–99.9%. The number of nucleotide substitutions varied between one and six among the nine 16S rRNA sequence types. One of the nine 16S rRNA sequence profiles was common to 12 of the strains from our study and two of these were identified as *Campylobacter coli *by PCR/REA. The other 10 strains were identified as *Campylobacter jejuni*. Five of the nine sequence types were also found among the *Campylobacter *sequences deposited in GenBank. The three 16S rRNA genes in the analysed strains were identical within each individual strain for all 47 strains.

**Conclusion:**

*C. jejuni *and *C. coli *seem to lack polymorphisms in their 16S rRNA gene, but phylogenetic analysis based on 16S rRNA sequences was not always sufficient for differentiation between *C. jejuni *and *C. coli*. The strains were grouped in two major clusters according to 16S rRNA, one cluster with only *C. jejuni *and the other with both *C. jejuni *and *C. coli*. Genotyping of the 47 strains by PFGE after digestion with *Sma*I resulted in 22 subtypes. A potential correlation was found between the *Sma*I profiles and the 16S rRNA sequences, as a certain *Sma*I type only appeared in one of the two major phylogenetic groups.

## Background

*Campylobacter *spp., principally *Campylobacter jejuni *subsp.*jejuni*, are important food- and water-borne pathogens for man [1-3]. In the present paper *Campylobacter jejuni *subsp. *jejuni *is referred to as *C. jejuni *and *Campylobacter jejuni *subsp. *doylei *as *C. doylei*. *Campylobacter jejuni *is frequently found in the intestinal tract in a wide variety of wild and domesticated animals, especially poultry [1,4]. The genus *Campylobacter *was introduced by Sebald and Verón [5] and its taxonomic structure has been revised a number of times [6-8]. At present the genus *Campylobacter *contains 17 species, four of which have been further divided into eight subspecies [9]http://www.bacterio.cict.fr/. The use of molecular methods in bacteriology has resulted in increased knowledge about biodiversity within the genus *Campylobacter*. Sequence analysis of the 16S rRNA gene has proved extremely useful for evolutionary studies of prokaryotes [10] and constitutes the basis for the revised taxonomy of bacteria [11]. This method has also been successfully used for identification of *Campylobacter *spp. [12]. Taxonomy has become a flexible and constantly evolving science and further progress in methodologies will probably result in additional adjustments in the classification of members of the genus *Campylobacter*. Furthermore, several PCR assays have successfully been applied for detection of *Campylobacter *with improved accuracy in identification of *Campylobacter *spp. from various sources.

Isolates from different bacteria can be identified to genus and often also to species level by sequence analysis of the 16S rRNA gene. Most species within the genus *Campylobacter *can be successfully differentiated by sequence analysis of the 16S rRNA gene. However, *C. jejuni*, *C. coli *and *C. lari *have been more difficult to differentiate by such sequence analysis [12]. Bacterial genomes can harbour up to 15 rRNA operons and the sequences of the corresponding genes are not always identical, which can make it difficult to interpret sequence data [13]. The nucleotide substitutions between different genes within a gene family are referred to as polymorphisms. Sequence differences can also occur between homologous genes from different strains of a certain bacterial species. All these nucleotide substitutions are collectively referred to as intraspecific variation. It has previously been reported that *C. jejuni *has three copies of the 16S rRNA gene in the genome [14,15]. One aim of our study was to establish if sequence differences exist between the three genes in a certain *Campylobacter jejuni *strain, because if intraspecific variation exists in these genes it may be possible to use the method for subtyping [16,17]. In a previous study of *Campylobacter *spp. from broilers in Sweden, 390 isolates were analysed by PFGE [18]. After digestion with *Sma*I, almost 80 different banding patterns (*Sma*I types) were obtained, including isolates that could not be digested with *Sma*I. Some of the subtypes were isolated more frequently than others. Another aim of the present study was to determine the sequence variation in the 16S rRNA genes of different *Campylobacter jejuni *strains and to determine whether the 16S rRNA sequence types correlated to the *Sma*I types.

## Materials and methods

### Bacterial strains

All bacterial strains were isolated from cloacal samples collected at slaughter within the Swedish Campylobacter programmes. The origins of the 47 strains (producer and slaughterhouse) are given in Table 1. The strains were isolated from different flocks delivered by 21 broiler producers to 6 different slaughterhouses during two periods, 1995–1997 and 2002–2004. First, two strains from each of the 10 most common *Sma*I types reported within the Campylobacter program in Sweden 2002–2004 were selected. Then eight new strains found at least four times in a previous Swedish study [18] were also selected for analysis. The selection was based on the *Sma*I profiles, and the profiles of these strains were similar to those of the 10 most common *Sma*I types. The 19 strains from 1995–1997 (Table 1) were chosen from the same producers as those from 2002–2004, which are known to frequently deliver *Campylobacter*-positive broilers [19]. The *Sma*I profiles are numbered according to previous studies in Sweden [18].

**Table 1:** Forty seven strains of *C. jejuni *and *C. coli *from cloacal samples taken within the Swedish *Campylobacter *programmes for broilers, for which the 16S rRNA gene sequences were determined in the present study

Isolate	16S rRNA seq. type	PFGE *Sma*I^1^	Year	Acc number^2^	Producer^3^	Slaughter- house^3^
13860/02	A	3	2002	EU127502	4	I
8266/02	A	3	2002	EU127503	7	II
13168/02	A	2	2002	EU127504	3	VI
3105-2/96	A	2	1996	EU127505	6	V
17903/02	B	4	2002	EU127506	20	III
18381/02	B	4	2002	EU127507	16	II
12739/02	B	10	2002	EU127508	10	V
10754/03	B	10	2003	EU127509	5	VI
1132-3/95	B	2	1995	EU127510	18	II
5169-1/96	B	2	1996	EU127511	4	I
5169-2/96	B	2	1996	EU127512	4	I
5174-2/96	B	2	1996	EU127513	4	I
6133-1/96	B	2	1996	EU127514	8	III
6188-1/96	B	2	1996	EU127515	7	III
11532/04	B	36	2004	EU127516	18	II
9612/04	B	42	2004	EU127517	10	V
10051/03	B	2	2003	EU127518	11	V
6113/97	B	2	1997	EU127519	20	III
6114/97	B	4	1997	EU127520	20	III
11036/96	C	2	1996	EU127521	19	IV
1160-2/96	D	10	1996	EU127522	16	II
1160-3/96	D	10	1996	EU127523	16	II
10056/03	E	5	2003	EU127524	15	II
10942/03	E	6	2003	EU127525	16	II
10713/03	E	8	2003	EU127526	14	V
11414/03	E	8	2003	EU127527	1	II
12717/02	E	9	2002	EU127528	2	III
3243/02	E	9	2002	EU127529	8	III
8693/04	E	67	2004	EU127530	18	II
11318/04	E	70	2004	EU127531	6	V
1167-3/95	E	101	1995	EU127532	18	II
1182-3/95	E	102	1995	EU127533	9	II
1184-3/95	E	102	1995	EU127534	9	II
8103/04	E	57	2004	EU127535	12	V
9582/04	F	26	2004	EU127536	19	V
10626/04	G	15	2004	EU127537	9	II
19280/02	H	1	2002	EU127538	21	I
18279/02	H	1	2004	EU127539	4	I
8525/02	H	5	2002	EU127540	17	I
12279/02	H	7	2002	EU127541	12	I
15193/03	H	7	2003	EU127542	8	III
5304/04	H	16	2004	EU127543	9	II
11020/96	H	20	1996	EU127544	19	IV
6144/96	H	5	1996	EU127545	2	III
6181/96	H	6	1996	EU127546	2	III
9916/03	H	6	2003	EU127547	17	I
11281/04	I	56	2004	EU127548	6	V

^1 ^The numbering of SmaI types 1 to 100 is according to other studies [15]. SmaI types assigned numbers greater than 100 have not been found previously in subtyping of isolates within the Swedish Campylobacter program 2001–2006.

^2 ^Accession number in GenBank.

^3 ^Origin of the isolates.

Procedures for sampling, transport and laboratory analyses have been described previously [19]. Identification of *Campylobacter *spp. was based on colony morphology, microscopic appearance and the following phenotypic characteristics: motility, production of cytochrome C oxidase and catalase, and the hippurate hydrolysis reaction [20]. One colony from each positive sample was stored in glycerol broth (15% glycerol and 85% serum broth) at -70°C before further use.

### Genotyping of selected strains by PCR/REA and PFGE

Species identification of *C. jejuni *was initially based on a positive hippurate hydrolysis reaction. Isolates that gave a weak or negative hippurate reaction were speciated by the polymerase chain reaction, followed by restriction enzyme analysis (PCR/REA) by which *C. jejuni, C. coli, C. lari*, and *C. upsaliensis *can be identified and differentiated [21]. In addition, some strains were tested by PCR for the hippuricase gene [22].

Genetic subtyping of *C. jejuni *isolates was performed by pulsed field gel electrophoresis (PFGE) in accordance with the standardised Campynet procedure [23]. Genomic DNA was digested with *Sma*I and the fragments were separated by PFGE in a CHEF-DRII apparatus (BioRad Laboratories, Hercules, CA, USA). The PFGE banding patterns were analysed by computer-assisted identification with GelCompar II (Applied Maths, Kortrijk, Belgium).

### Sequencing of 16S rRNA genes

DNA was prepared from the bacteria by lysis of 1–3 colonies in 100 μl of water at 100°C for 10 min. The lysate was used as a template for in vitro amplification of the 16S rRNA genes by PCR. The PCR products were used for cycle sequencing with fluorescently labelled terminators (Big Dye; Applied Biosystems, Foster City, Calif., USA) as described by the manufacturer and with a set of sequencing primers developed for members of the phylum *Proteobacteria *[24]. The sequencing products were analysed by capillary electrophoresis on an ABI Prism 3100 genetic analyser (Applied Biosystems). Contiguous sequences (contigs) were generated by using the program Contig Express included in the Vector NTI Suite (InforMax, Bethesda, Md., USA). The contigs were checked and edited manually if necessary before phylogenetic analysis and deposition in GenBank. The accession numbers for the sequences in GenBank are given in Table 1.

### Phylogenetic analysis

The 16S rRNA sequences determined in this work were aligned manually with prealigned sequences retrieved from the Ribosomal Database Project II [25] and by using the Genetic Data Environment software [26]. The phylogenetic trees were constructed by neighbour-joining [27] from a distance matrix that was corrected for multiple substitutions at single locations by the two-parameter method [28]. The distance matrix comprised 1417 nucleotide positions corresponding to positions 36 to 1472 in the 16S rRNA sequence of *Escherichia coli*.

## Results

### Identification of the isolates

Forty-five of the 47 strains included in this study were identified as *Campylobacter jejuni *by a positive hippurate hydrolysis reaction or by PCR/REA or by the presence of the hippuricase gene as judged from PCR. Two strains were identified as *C. coli *(8693/04 and 11318/04) by the same methods. All isolates were identified as representing either *C. jejuni *or *C. coli *by 16S rRNA sequence analysis.

### Sequence analysis of the 16S rRNA genes

**Figure 1:**
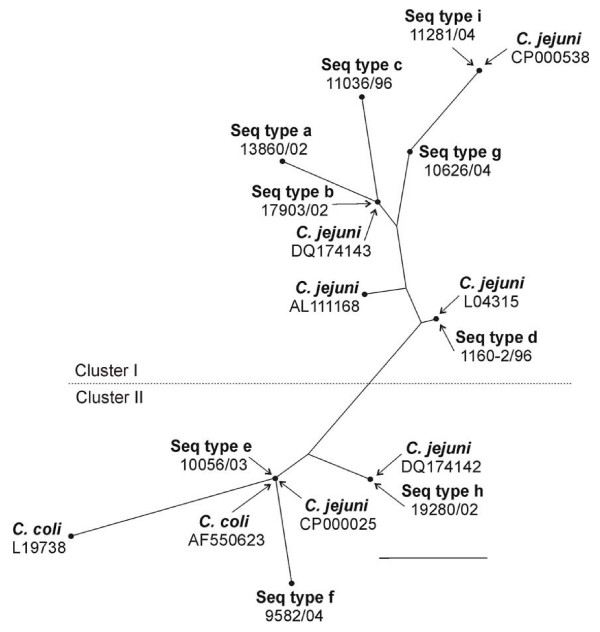
Radial representation of a phylogenetic tree constructed by the neighbour-joining method showing the relationships between the different sequence types (Seq type), *C. coli *and *C. jejuni*. *Campylobacter coli *was used as outgroup. The length of the scale bar represents one nucleotide substitution in the 16S rRNA gene fragment (1417 nucleotides).

The length of the 16S rRNA gene fragments of the sequenced *Campylobacter *isolates was 1417 nucleotides. Ambiguities were not found in any of the sequences, which showed that there were no sequence polymorphisms in the three 16S rRNA genes. However, nucleotide substitutions were found in eight positions of the strains sequenced in the present study, which resulted in nine different 16S rRNA sequence types, referred to as a-i in Tables 1, 2 and 3. The nucleotide sequences of the 16S rRNA genes of the 47 strains were determined and compared with 16S rRNA sequences of 21 strains of *C. jejuni*, 2 strains of *C. doylei*, one strain each of *C. lari *and *C. upsaliensis *and 16 strains of *C. coli *retrieved from RDP-II (Table 2). Nine additional positions that were variable were identified in the sequences deposited by other authors (Table 3). The Swedish strains were not variable in these positions. The sequence similarities between the nine different sequence types (a-i) were in the range 99.6–99.9%. The sequence similarities among *C. jejuni *strains and among *C. coli *strains were in the range 99.5–99.9% and 98.2–99.9%, respectively. Whole genome sequences are available for four of the *C. jejuni *strains (accession numbers, CP000025, AL111168, CP000814 and CP000538). Whole genome sequence data show that the genomes of *Campylobacter jejuni *strains harbour three rRNA operons and we found that the sequences of the three 16S rRNA genes were identical within each individual strain for all 47 strains. Furthermore, the three 16S rRNA genes of *C. doylei *(CP000768) are also identical. The nine profiles occurred with varying frequencies among the 47 strains analysed here. Three 16S rRNA sequence types (b, e and h) dominated and constituted 79% (37 of 47 strains). The number of nucleotide substitutions varied between one and six among the nine 16S rRNA sequence types. The largest difference was found between the 16S rRNA sequence types f and i (see Fig. 1). Furthermore, five of the sequence types (b, d, e, h and i) had identical 16S rRNA sequence profiles to strains retrieved from GenBank. Sequence type (e) was shared between 12 of the strains in our study and two of these were identified as *C. coli *by PCR/REA. These strains had an identical 16S rRNA profile to *C. jejuni *(CP000025), for which the whole genome sequence is available, and also to one strain of *C. coli *(AF550623).

**Table 2:** *Campylobacter *spp. strains for which the 16S rRNA sequences were retrieved from RDP-II or GenBank and used in the phylogenetic analysis

Species	Strain	Acc no in GenBank	Acc no to an identical seq	No of Ns^4 ^in the seq	Identical to Seq type
*C. coli*	LMG 6440	AF372092			
*C. coli*	LMG 9220	AF550620			
*C. coli*	LMG 15883	AF550621			
*C. coli*	LMG 15884	AF550622			
*C. coli*	H99/155	AF550624			
*C. coli*	B99/131	AF550625			
*C. coli*	ATCC 49941	AY621115			
*C. coli*	NZ1905-94	DQ174136			
*C. coli*	NZ2695-96	DQ174137			
*C. coli*	CCUG 11283	L04312		10	
*C. coli*	RMIT32A	L19738		12	
*C. coli*	H99/119	AF550623			e
*C. coli*	Lio8	DQ174135^3^	AF550623		e
*C. coli*	NZ899-00	DQ174138^3^	AF550623		e
*C. coli*	NZ900-95	DQ174139^3^	AF550623		e
*C. coli*	NZ4812-94	DQ174140^3^	AF550623		e
*C. jejuni*	RM1221^2^	CP000025			e
*C. jejuni*	98/E600/5	AF393202^3^	RM1221		e
*C. jejuni*	98/E599/10	AF393203^3^	RM1221		e
*C. jejuni*	LMG9217	AF550626^3^	RM1221		e
*C. jejuni*	CCUG 10937	DQ174141^3^	RM1221		e
*C. jejuni*	NCTC 11351	AF372091			
*C. jejuni*	CCUG 11284	L04315		9	
*C. jejuni*	WH11	AF393204^3^	L04315		d
*C. jejuni*	BB/224	AF550629^3^	L04315		d
*C. jejuni*	LMG 9243	AF550627			
*C. jejuni*	H99/240	AF550628			
*C. jejuni*	TGH9011	Z29326			
*C. jejuni*	SSI 5384-98	Y19244			
*C. jejuni*	NCTC 11168^1,2^	AL111168			
*C. jejuni*	6871	AY628389			
*C. jejuni*	81–176^2^	CP000538			i
*C. jejuni*	ATCC 294228	DQ174142			h
*C. jejuni*	B99/206	AF550630^3^	DQ174142		h
*C. jejuni*	Lio6	DQ174143			b
*C. jejuni*	ATCC 49943	AY621112^3^	DQ174143		b
*C. jejuni*	CCUG 24567	L14630^3^	DQ174144		
*C. doylei*	LMG8843	DQ174144			
C. doylei	Not defined	AY621111^3^	DQ174143		
*C. lari*	Not defined	L04316		6	
*C. upsaliensis*	Not defined	L14628		11	

^1 ^Strain for which the whole genome sequence is available.

^2 ^The sequence without deletions was used in the tree.

^3 ^This sequence was not used in the tree (Fig. 2), because it was identical to another sequence.

^4 ^Number of ambiguities in the sequence, which also reflects the sequencing accuracy.

**Table 3:** Polymorphic positions in the nine different 16S rRNA sequence types obtained from *C. jejuni *isolated from cloacal samples taken within the Swedish *Campylobacter *program for broilers.

Seq type	No of isolates	80	126	141*	205	554*	614*	644	687*	703*	712*	814	821*	986	995	1228*	1244	1400*
A	4	C	A	C	C	C	G	C	C	G	C	A	T	A	T	C	C	T
B	15	C	A	C	T	C	G	C	C	G	C	A	T	A	T	C	C	T
C	1	C	A	C	T	C	G	C	C	G	C	A	T	A	T	C	T	T
D	2	C	A	C	T	C	G	C	C	G	C	G	T	A	T	C	C	T
E	12	C	A	C	T	C	G	C	C	G	C	G	T	T	A	C	C	T
F	1	C	G	C	T	C	G	C	C	G	C	G	T	T	A	C	C	T
G	1	T	A	C	T	C	G	C	C	G	C	A	T	A	T	C	C	T
H	10	T	A	C	T	C	G	C	C	G	C	G	T	T	A	C	C	T
i	1	T	A	C	T	C	G	T	C	G	C	A	T	A	T	C	C	T
CP000025	1	C	A	C	T	C	G	C	C	G	C	G	T	T	A	C	C	T
AL111168	1	T	A	C	T	C	G	C	C	G	C	G	T	A	T	C	C	T

*Positions where some of the C. jejuni 16S rRNA sequences deposited in GenBank by other authors differed from the sequences determined in this study

The nucleotide positions were numbered according to one of the 16S rRNA gene sequences of *C. jejuni *strain NCTC 11168, for which the whole genome sequence is available from GenBank. However, the 16S rRNA sequence was retrieved from RDP-II [29].

### Phylogenetic analysis

**Figure 2:**
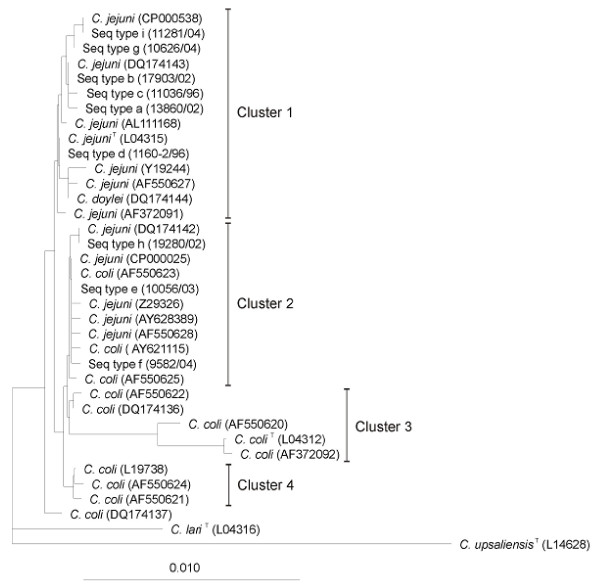
Evolutionary tree showing the phylogenetic relationships between *C. lari*, *C. doylei*, *C. coli *and *C. jejuni *retrieved from RDP-II and the nine sequence types (Seq type) of *Campylobacter *spp. identified in this study. A representative strain is given in brackets after the sequence types. Accession numbers in GenBank are given for the other strains. *Camplyobacter upsaliensis *was used as outgroup. The length of the scale bar represents 1 nucleotide substitution per 100 positions.

The strains in the present study were grouped into two major clusters in the radial tree (Fig. 1). The first cluster comprised sequence types a, b, c, d, g and i and contained sequence types of *C. jejuni *retrieved from GenBank (Fig. 2). The sequence types a, b, c, g and i had the nucleotides A, A and T in the positions 814, 986 and 995, respectively, whereas sequence type d had a G in the first of these positions (Table 3). The *Sma*I types in this group were 2, 3, 4, 10, 15, 36, 42 and 56. The second cluster comprised sequence types e, f and h and contained both *C. coli *and *C. jejuni*. These strains are characterised by having G, T, and A in nucleotide positions 814, 986 and 995, respectively (Table 3). The corresponding *Sma*I types in this group were 1, 5, 6, 7, 8, 9, 16, 20, 26, 57, 67, 70, 101 and 102.

Phylogenetic relationships of the nine 16S rRNA sequence types obtained in the present study and certain strains of *C. jejuni*, *C. coli*, *C. doylei, C. lari *and *C. upsaliensis *reported by other authors are shown in Fig. 2. The taxa in this tree form four clusters (1–4) and one single species line (*C. coli*, DQ174137). However, due to the high-sequence similarity, the nodes are not very stable and sequencing errors in some of the older sequences may well affect the topology of the tree. Cluster 1 contains only *C. jejuni *and *C. doylei *strains. Cluster 2 contains both *C. jejuni *and *C. coli *strains, while clusters 3 and 4 contain only *C. coli *strains. The three *C. coli *strains (AF550620, L04312 and AF372092) have sequence similarities to *C. jejuni *in the range 98.3–98.6%.

### Pulsed-field gel electrophoresis

**Figure 3:**
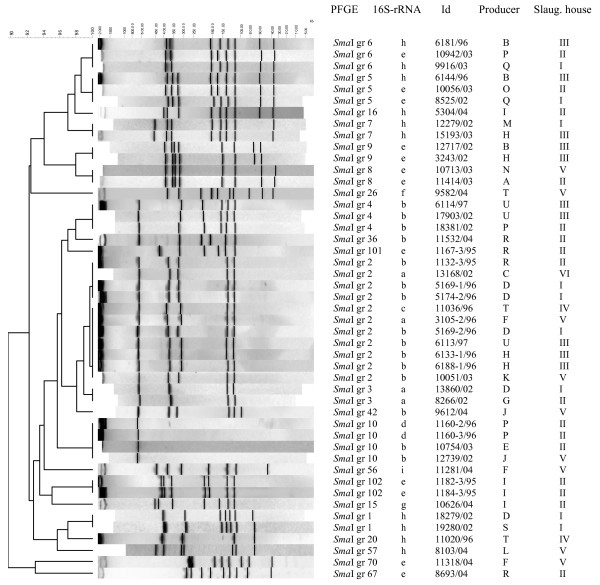
Dendrogram of the PFGE profiles obtained after *Sma*I digestion of DNA from *Campylobacter jejuni *and *C. coli *strains from cloacal samples taken within the Swedish Campylobacter program for broilers. The numbering of *Sma*I types 1 to 100 is according to other studies [15]. *Sma*I types assigned numbers greater than 100 have not been found previously in subtyping of isolates within the Swedish Campylobacter program.

The 47 strains were subtyped into 22 different *Sma*I types, including the strains refractory to *Sma*I digestion (designated *Sma*I type 10). Of the 22 *Sma*I types, 11 were found only once, and the remaining 11 subtypes were found at least twice (Fig. 3). The subtype that was most often observed was *Sma*I type 2 (11 isolates) and this was also a subtype that was widely spread among producers, slaughterhouses and years. No correlation could be found between the different 16S rRNA sequences or *Sma*I types and the year, slaughterhouse or farm from which the *Campylobacter *strain had been isolated. Strains with the same *Sma*I type but of different sequence types appeared only in one the two phylogenetic clusters (Fig 1).

## Discussion

Genomes of *C. jejuni *and *C. coli *only harbour three copies of the 16S rRNA genes, whereas *E. coli *has seven rRNA genomic loci [14,29,30]. The three 16S rRNA gene sequences of the respective 47 strains included in the study were identical as judged from the sequence raw data, which would have revealed any polymorphism. This finding was surprising because bacteria with more than one rRNA operon often have at least a few polymorphisms in their 16S rRNA genes. The observation that *C. jejuni *seem to lack polymorphisms in their 16S rRNA genes is also supported by genome sequencing data, because three of the strains (CP000025, CP000814 and AL111168) have identical 16S rRNA genes. The third strain (CP000538) has two deletions in one of the three genes, but except for that, the three genes are also identical. We did not find sequence length polymorphisms and in this respect *C. jejuni *is very stable. Reparation mechanisms for concerted evolution of rRNA genes seem to be very efficient. Furthermore, the whole genome sequence of *Campylobacter doylei *(CP000768) also contains three identical 16S rRNA sequences.

It was possible to identify eight signature nucleotide positions by which the studied strains of *C. jejuni *could be differentiated into nine groups according to their 16S rRNA sequences. The observed nucleotide substitutions at different positions in the 16S rRNA genes of *Campylobacter *spp. were compared with the positions of the nucleotide substitutions that appear as mutations in *E. coli *[31]. However, none of the nucleotide substitutions was found at the homologous positions. Restriction analysis by *Sma*I digestion and PFGE was, however, a more discriminatory typing method, detecting 22 subtypes as compared with nine different 16S rRNA sequence types. The discriminatory power of PFGE for genotyping of *Campylobacter *spp. has been reported previously [32,33]. One sequence type (e) had identical 16S rRNA sequence as one of the whole genome sequenced *C. jejuni *strains (RM1221) and *C. coli *strain (Lio8), (Table 2, Fig 2). Furthermore, sequence type e differed in only two nucleotide positions from *C. coli *(L19738). Two of the isolates (8693/04 and 11318/04) belonging to16S rRNA type e were also identified as *C. coli *by PCR/REA. This observation shows that phylogenetic analysis based on 16S rRNA cannot always be used to differentiate between *C. jejuni *and *C. coli*, although in cluster 3 (Fig. 2), three of the *C. coli *sequences are significantly different from the *C. jejuni *sequences. However, the results must be interpreted with care, because sequences of many mistyped bacterial strains have been deposited in GenBank. The results in the present study confirm the limitations of the 16S rRNA gene for resolving close, relationships.

The common *Sma*I types identified in Swedish broilers in 2002–2004 were also found among isolates from 1995–1997, a fact that indicates that these common types are also stable over time. Furthermore, the whole genome sequenced *C. jejuni *(CP000025) with an identical profile to sequence type e originates from chickens in the USA [34]. This raises the question whether certain clones are more common than others and whether those clones have a higher ability to survive in the environment around the broilers or within animals around the broiler houses over a very long time. Bacteria with relatively small genomes, such as *C. jejuni*, may undergo genetic variation to increase their potential to adapt to new environments [14]. Such genotypic variation could result in phenotypic changes. These variations are probably important in the transmission route from broiler to man, where *Campylobacter *spp. must survive several hostile environments.

## Conclusion

Bacterial genomes could harbour up to fifteen 16S rRNA operons. Polymorphisms in the 16S rRNA gene are common in bacteria with more than one 16S rRNA operon. Genomes of *Campylobacter *spp. harbour three copies of the 16S rRNA genes. Polymorphisms were not found in the 16S rRNA genes from any of the 47 *Campylobacter *spp. strains sequenced. The three rRNA operons in the analysed *Campylobacter jejuni *strains were identical within each individual strain for all 47 strains, which indicates that *C. jejuni *and *C. coli are *very stable in this respect.

Genotyping of 47 strains by 16S rRNA gene sequencing resulted in nine sequence types, whereas PFGE after digestion with *Sma*I resulted in 22 subtypes. Phylogenetic analysis based on 16S rRNA sequences is not always sufficient for differentiation between *C. jejuni *and *C. coli*. A potential correlation was found between the *Sma*I profiles and the 16S rRNA sequences, as a certain *Sma*I type only appeared in one of the two major phylogenetic groups.

## Authors' contributions

IH, EOE and KEJ participated in the discussion on the study design, the collection of isolates, analysis and interpretation of data, and in the writing of the manuscript, EOE and LS carried out the analysis and interpretation of PFGE data. Analysis and interpretation of sequence data of the 16S rRNA gene were carried out by IH, MP and KEJ. All authors read and approved the final manuscript.

## References

[B1] Skirrow MB (1994). Diseases due to *Campylobacter*, *Helicobacter *and related bacteria. J Comp Pathol.

[B2] Bryan FL, Doyle MP (1995). Health risks and consequences of *Salmonella *and *Campylobacter jejuni *in raw poultry. J Food Prot.

[B3] Altekruse SF, Stern NJ, Fields PI, Swerdlow DL (1999). *Campylobacter jejuni *– an emerging foodborne pathogen. Emerg Infect Dis.

[B4] Blaser MJ, Taylor DN, Feldman RA (1983). Epidemiology of *Campylobacter jejuni *infections. Epidemiol Rev.

[B5] Sebald ER, Véron M (1963). DNA base content and classification of vibrios (In French; Teneur en bases da lÀDN et classification des Vibrions). Annales de L'institut Pasteur (Paris).

[B6] Goodwin CS, Armstrong JA, Chilvers T, Peters M, Collins MD, Sly L, McConnell W, Harper WES (1989). Transfer of *Campylobacter pylori *and *Campylobacter mustelae *to *Helicobacter *gen. nov. as *Helicobacter pylori *comb. nov. and *Helicobacter mustelae *comb. nov., respectively. J Syst Bact.

[B7] Vandamme P, Falsen E, Rossau R, Hoste B, Segers P, Tytgat R, De Ley J (1991). Revision of *Campylobacter*, *Helicobacter*, and *Wolinella *taxonomy: Emendation of generic descriptions and proposal of *Arcobacter *gen. nov. Int J Syst Bacteriol.

[B8] Vandamme P, On SLW (2001). Recommendations of the subcommittee on the taxonomy of *Campylobacter *and related bacteria. Int J Syst Evol Microbiol.

[B9] List of procaryotic names with standing in Nomenclature. http://www.bacterio.cict.fr/.

[B10] Woese CR (1987). Bacterial evolution. Microbiol Reviews.

[B11] Ludwig W, Klenk HP, Boone DR, Castenholz RW (2001). A phylogenetic backbone and taxonomic framework for procaryotic systematics. Bergey's Manual of Systematic Bacteriology The Archaea and the Deeply Branching and Phototrophic Bacteria.

[B12] Gorkiewicz G, Feierl G, Schober C, Dieber F, Köfer J, Zechner R, Zechner EL (2003). Species-specific identification of campylobacters by partial 16S rRNA gene sequencing. J Clin Microbiol.

[B13] Parkhill J, Wren BW, Mungall K, Ketley JM, Churcher C, Basham D, Chillingworth T, Davies RM, Feltwell T, Holroyd S, Jagels K, Karlyshev AV, Moule S, Pallen MJ, Penn CW, Quail MA, Rajandream MA, Rutherford KM, van Vliet AH, Whitehead S, Barrell BG (2000). The genome sequence of the food-borne pathogen *Campylobacter jejuni *reveals hypervariable sequences. Nature.

[B14] Taylor DE, Eaton M, Yan W, Chang N (1992). Genome maps of *Campylobacter jejuni *and *C. coli*. J Bacteriol.

[B15] Kim NW, Lombardi R, Bingham H, Hani E, Louie H, Ng D, Chan VL (1993). Fine mapping of the three rRNA operons on the updated genomic map of *Campylobacter jejuni *TGH9011 (ATCC 43431). J Bacteriol.

[B16] Heldtander Königsson M, Bölske G, Johansson KE (2002). Intraspecific variation in the 16S rRNA gene sequences of *Mycoplasma agalactiae *and *Mycoplasma bovis *strains. Vet Microbiol.

[B17] Heldtander M, Wesonga H, Bölske G, Pettersson B, Johansson KE (2001). Genetic diversity and evolution of *Mycoplasma capricolum *subsp. *capripneumoniae *strains from eastern Africa assessed by 16S rDNA sequence analysis. Vet Microbiol.

[B18] Hansson I, Vågsholm I, Svensson L, Engvall EO (2007). Correlations between *Campylobacter *spp. prevalence in the environment and broiler flocks. J Appl Microbiol.

[B19] Hansson I, Engvall EO, Lindblad J, Gunnarson A, Vågsholm I (2004). The *Campylobacter *surveillance program for broilers in Sweden, July 2001–June 2002. Vet Rec.

[B20] Nachamkin I, Murray PR, Baron EJ, Pfaller MA, Tenover FC, Yolken RH (1995). *Campylobacter *and *Arcobacter*. Manual of Clinical Microbiology.

[B21] Fermér C, Engvall EO (1999). Specific PCR identification and differentiation of the thermophilic campylobacters, *Campylobacter jejuni, C. coli, C. lari*, and *C. upsaliensis*. J Clin Microbiol.

[B22] Linton D, Lawson AJ, Owen RJ, Stanley J (1997). PCR detection, identification to species level, and fingerprinting of *Campylobacter jejuni *and *Campylobacter coli *direct from diarrheic samples. J Clin Microbiol.

[B23] Standardised Campynet procedure. http://www.svs.dk/campynet/PFGE.html.

[B24] Båverud V, Nyström C, Johansson KE (2006). Isolation and identification of *Taylorella asinigenitalis *from the genital tract of a stallion, first case of a natural infection. Vet Microbiol.

[B25] Cole JR, Chai B, Farris RJ, Wang Q, Kulam SA, McGarrell DM, Garrity GM, Tiedje JM (2005). The Ribosomal Database Project (RDP-II): Sequences and tools for high-throughput rRNA analysis. Nucleic Acids Res.

[B26] Smith S (1992). GDE: Genetic Data Environment. Version 22 Millipore Imaging Systems.

[B27] Saitou N, Nei M (1987). The neighbour-joining method: A new method for reconstructing phylogenetic trees. Mol Biol Evol.

[B28] Kimura M (1980). A simple method for estimating evolutionary rates of base substitutions through comparative studies of nucleotide sequences. J Mol Evol.

[B29] Nuijten PJM, Bartels C, Bleumink-Pluym C, Gaastra W, Zeijst BAM van der (1990). Size and physical map of the *Campylobacter jejuni *chromosome. Nucleic Acids Res.

[B30] Kim NW, Bingham H, Khawaja R, Louie H, Hani E, Neote K, Chan VL (1992). Physical map of *Campylobacter jejuni *TGH9011 and localisation of 10 genetic markers by use of pulsed-field gel electrophoresis. J Bacteriol.

[B31] Triman KL (1996). The 16S ribosomal RNA mutation database (16SMDB). Nucleic Acids Res.

[B32] Wassenaar T, Geilhausen B, Newell D (1998). Evidence of genomic instability in *Campylobacter jejuni *isolated from poultry. Appl Environ Microbiol.

[B33] de Boer P, Duim B, Rigter A, Plas J van der, Jacobs-Reitsma WF, Wagenaar JA (2000). Computer-assisted analysis and epidemiological value of genotyping methods for *Campylobacter jejuni *and *Campylobacter coli*. J Clin Microbiol.

[B34] Parker CT, Quinones B, Miller WG, Horn ST, Mandrell RE (2006). Comparative genomic analysis of *Campylobacter jejuni *strains reveals diversity due to genomic elements similar to those present in *C. jejuni *strain RM1221. J Clin Microbiol.

